# Christmas Festivities: Their Helpfulness and Injustice

**Published:** 1912-12-21

**Authors:** 


### HOSPITAL WEAKNESS AND HOSPITAL STRENGTH.

(Contributions to this page will be welcomed.)

### IV. Christmas Festivities: Their Helpfulness and Injustice.

We have held for thirty years at least that no one of intelligence who wants to learn something of the life of great cities from every point of view, and to raise their own standard of personal conduct to the highest point, should fail to spend some hours on Christmas Day in a great voluntary hospital. It has been our practice to pursue this course in order to show our sympathy with all the workers, rather than with the patients, for Christmas Day in one of the voluntary hospitals, for some surprising reason we have never been able quite to account for, is for many of the patients the day of their lives. On the first blush it might be thought that patients who were seriously ill would wish for solitude and quietness on Christmas Day and every day till convalescence comes. Our observation, which extends over many years in many hospitals, large and small, convinces us that, speaking of the patients as a whole, however ill they may be, Christmas Day in a hospital is usually a time of infinite pleasure, exhilaration, and thankfulness. Christmas in a British hospital is an occasion which every man and woman of intelligence should use to make themselves familiar with the inside of such an institution. The occasion would give them a unique opportunity for personal contact with the work and with the spirit in which that work is done. It may therefore be assumed that the Christmas festivities in the voluntary hospitals are welcomed by and good for the patients.

But what of the workers? Those who adopt our suggestion and pay a visit on Christmas Day to a voluntary hospital, especially if it has a medical school attached, will probably find there the most brilliant decorations. These are the outcome of every effort which the energy of youth and the best instincts of early manhood can put forth to cheer the patients and make them happy and forgetful of their sufferings. The humane influence which is being exercised upon society through these efforts must be apparent to every visitor. The outside world little realises the beauties which the combined work of students, nurses, and medical staff introduces to the hospital wards at Christmas time. Every hospital is a small republic in its way, and each ward under a separate staff is as zealous for its reputation at this season as the haughtiest State on the earth's surface. It would be invidious to make comparisons, but, judging by many years' experience, we can confidently say that the results in the wards of, say, Guy's Hospital, for example, will be found to be so excellent as to be worthy of the critical inspection of artists generally, and especially of those who devote themselves to the illustration of Christmas literature. The infinite variety of design, not only in the character but in the detail of the decorations is remarkable. Let a visitor take notice of the many separate designs in the manufacture of ropes of evergreens which the wards may contain. Note, too, those wards which are remarkable for the beauty of their flowers and the atmosphere of hospitality which every sister lays herself out to offer to the visitors who come from outside to help in the good work of making Christmas Day happy and pleasant to the sick. Few, if any, family gatherings anywhere produce more happiness or exercise a wider influence for good than those to be found in many hospital wards. Everybody on these occasions is so friendly and pleasant that all feel at home and at peace.

These results have been achieved by the resident staff mainly, who not only have devoted the whole of their leisure, for many days before Christmas, to preparation, but who often expend an amount of money upon the decorations out of all proportion to their slender means. Yet such gifts, in fact, in the givers' opinion, may be far too small a proportion of the generosity of their hearts. The sisters and nurses, from daylight till long into the evening, willingly devote themselves to the promotion of happiness and cheerfulness, for cheerfulness in the sick is the high road to recovery. Who can wonder if the habit of hospital patients in the old days to go home for Christmas, is now, especially among the children of the poor, changed to a desire to be a resident in a voluntary hospital during Christmas week?

All this loving sacrifice testifies to the humane influence of the voluntary hospitals, and we can but hope, as it becomes better realised by the governors and outside public, that the whole cost involved by the Christmas festivities will be spontaneously provided from these sources. It is not right that a large portion of the cost, or, indeed, any portion of it, should be borne by the sisters or the nurses in any hospital. We should like to see a new system introduced by the management which would invite a small committee of governors to interest themselves in the Christmas festivities a month beforehand and make themselves responsible for providing the, to them, relatively small cost entailed. We have tried this plan and have found it work admirably. It would, at any rate, obviate the injustices which are pointed out by Mr. James L. Birley, of the National Hospital, in *St. Thomas' Hospital Gazette* for December. Few, if any, festivities can be made really enjoyable without music, both vocal and instrumental. It is the students who usually provide this important addition to the patients' happiness. Mr. Birley brings this point out clearly by stating that the Pierrot troupes often experience a lack of funds, and that these deficiencies have had to be made good for years out of the pockets of the students. Neither sisters, nurses, nor students ought to have to contribute anything to such expenses. We hope by calling attention to this injustice to stir up at least one governor amongst our readers at every voluntary hospital who will call at once on the secretary or matron of the hospital in his

* Previous articles in this series have appeared in our issues of Nov. 2, 16, 30.

neighbourhood, and offer to defray the cost of the whole or a portion of its Christmas expenses. Personal service is a glorious thing to engage in. But when rendered in the right spirit it is epoch-making for all concerned. Even those who cannot or do not pursue this service can give an infinity of joy and inspiration to the workers by spontaneously showing practical sympathy with them in the way here suggested. So the blessedness of Christmas Day in the voluntary hospitals may be shared by a large number of people this year who read this article, if they are warm-hearted and vigorous enough to welcome with delight so novel an experience.

